# Formation of sub-100-nm suspended nanowires with various materials using thermally adjusted electrospun nanofibers as templates

**DOI:** 10.1038/s41378-022-00459-y

**Published:** 2023-02-17

**Authors:** Yongkeun Oh, Dae-Sung Kwon, Eunhwan Jo, Yunsung Kang, Sangjun Sim, Jongbaeg Kim

**Affiliations:** School of Mechanical Engineering, Yonsei University, Seoul, 03722 Republic of Korea

**Keywords:** Nanowires, Nanosensors

## Abstract

The air suspension and location specification properties of nanowires are crucial factors for optimizing nanowires in electronic devices and suppressing undesirable interactions with substrates. Although various strategies have been proposed to fabricate suspended nanowires, placing a nanowire in desired microstructures without material constraints or high-temperature processes remains a challenge. In this study, suspended nanowires were formed using a thermally aggregated electrospun polymer as a template. An elaborately designed microstructure enables an electrospun fiber template to be formed at the desired location during thermal treatment. Moreover, the desired thickness of the nanowires is easily controlled with the electrospun fiber templates, resulting in the parallel formation of suspended nanowires that are less than 100 nm thick. Furthermore, this approach facilitates the formation of suspended nanowires with various materials. This is accomplished by evaporating various materials onto the electrospun fiber template and by removing the template. Palladium, copper, tungsten oxide (WO_3_), and tin oxide nanowires are formed as examples to demonstrate the advantage of this approach in terms of nanowire material selection. Hydrogen (H_2_) and nitrogen dioxide (NO_2_) gas sensors comprising palladium and tungsten oxide, respectively, are demonstrated as exemplary devices of the proposed method.

## Introduction

The productivity of a fabrication method and the uniformity of fabricated devices are important factors for commercialization^1–3^. Micro-electro-mechanical system (MEMS) technology has offered high productivity and uniformity; thus, it is widely used for commercialization. This technology is currently being used to produce many types of microscale sensors and actuators. For example, miniaturized MEMS devices are used in smartphones, cars, factories, and clothing^4–7^. Owing to the increase in demand for these devices, smaller sizes and better performance are often required, and research on MEMS devices using nanomaterials is being conducted as an attractive method to improve performance^8–17^.

Nanomaterials exhibit unique characteristics owing to their small sizes, and in many cases, their performance is better than bulk-sized materials when used as a sensing material^11–17^. However, the small sizes of nanomaterials affect the uniformity and productivity of manufacturing devices. Among these nanomaterials, the performance of nanowires used in gas sensors varies depending on the length and thickness, and it is difficult to specify where they are formed. In particular, suspended nanowires have a higher surface-to-volume ratio than those attached to a substrate and thus typically show better sensing performance owing to the lack of thermal and electrical interactions with substrates^18–21^. However, the fabrication of suspended nanowires requires a more complicated process.

Electrospinning is a conventional production method among several fabrication approaches for suspended nanowires^22–29^. In typical electrospinning, a jet with whipping instability is generated by an electric force, and the polymer solution travels to the target substrate. During travel, the solvent evaporates, thereby forming solid polymer fibers on the target substrate. The characteristics of the electrospinning process in which polymer fibers are randomly dispersed have been studied for fabricating nanonetworks^30,31^. Moreover, when electrospinning is performed on a microstructure, polymer fibers are formed in the upper part of the step, facilitating the fabrication of suspended nanowires. However, controlling the number, length, and thickness of nanowires is difficult owing to random dispersion characteristics. Therefore, the method for fabricating nanowires only in specific locations through a lithography technique using a mask on an electrospun SU-8 fiber has been studied; however, the fabricated nanowires were limited to photoresist materials^32,33^.

Another fabrication method for suspended nanowires with location control is the direct growth of nanowires on a microstructure. This method can control the position of nanowires by patterning catalysts in a selective area^34,35^. However, the number of nanowires formed through this method is not accurately controlled; thus, no single nanowire can be fabricated. Other studies have also been conducted using lithography. In one of the studies, photoresist structures containing suspended wires were produced by adjusting the light intensity during lithography. Thereafter, the photoresist wire was converted into a nanoscale carbon nanowire through calcination^22,23^. This method is capable of batch fabrication, and it improves the uniformity of the fabricated nanowires; nevertheless, the material selection of the fabricated nanowires is limited to carbon. Other studies using lithography have been conducted to fabricate nanolene in a C-shaped channel^36^. This method involves fabricating nanowires with a C-shaped cross-section on a nanograting MEMS substrate that is prepared through angled evaporation twice, and then the MEMS substrate is removed using XeF_2_ dry etching. This method allows nanowires to be formed with various materials deposited by evaporation in an array. However, this fabrication process involves several expensive steps performed in vacuum chambers, and the minimum size of the fabricated nanowires is limited to at least 100 nm.

Here, we propose a fabrication method for suspended nanowires with controlled locations and numbers. The main mechanism of the proposed method is that thermally treated polymer fibers migrate to a more stable location. We specified the stable locations through the microstructure design and formed polymer fibers on it through electrospinning. Afterward, thermal treatment was conducted to ensure that the electrospun polymer fibers were aggregated at the desired location. The desired material was deposited using these aggregated polymer wires as a template, and then the polymer was removed. Through this process, nanowires of the desired material were formed at the desired location. All techniques used in the fabrication of the nanowires were inexpensive, and batch fabrication was possible. Furthermore, the characteristics of the fabrication method, such as thickness, length, location, and the number of nanowires, can be controlled.

The number and length of nanowires that are formed can be controlled by designing the microstructure, and the thickness of the nanowires can be controlled by adjusting the electrospinning conditions and reducing the size of the aggregated polymer wires. Furthermore, the materials of the nanowires are not particularly limited if they can be deposited by evaporation. We fabricated copper, palladium, tungsten oxide (WO_3_), and tin oxide (SnO_2_) nanowires using the developed method. Among them, Pd and WO_3_ nanowires are available directly as gas sensors after simple wire bonding. The Pd and WO_3_ nanowires were tested for H_2_ and NO_2_ gas sensing, respectively, through which the electrical characteristics of the fabricated nanowires were verified, and they were considered suitable for applications.

## Results

### Fabrication process for suspended nanowires

**Fig. 1:**
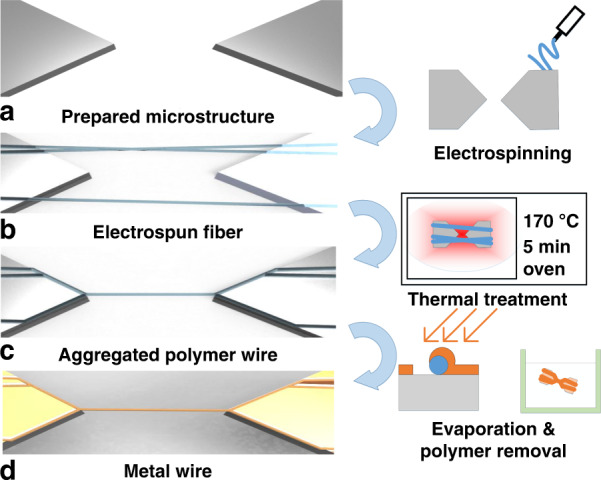
Schematics for nanowire fabrication methods using thermal treatment. The process consists of electrospinning, thermal treatment, evaporation, and a wet process. **a** The microstructure was prepared by general Silicon on insulator (SOI) MEMS fabrication, **b** polymer fibers were formed by electrospinning, **c** polymer fibers were aggregated to a single polymer wire at the tip end during thermal treatment, and **d** the polymer wire was exchanged to other materials by evaporation and a wet process

Figure 1 shows a conceptual design of the fabrication process using thermal treatment. A polymer wire is formed and then replaced with another material throughout the fabrication process. The properties of the preformed polymer wire determine the characteristics of the nanowire. The fabrication process of the polymer wire consists of microstructure preparations, electrospinning, and thermal treatment. A proper design is required because the microstructure affects the migration of polymer fibers during thermal treatment (Fig. 1a). Regarding the microstructure, a step in which the electrospun fibers are typically produced in a suspended form should be included, although the details may vary depending on the design. After forming a random number of polymer fibers through the electrospinning process (Fig. 1b), thermal treatment was applied using ovens or hot plates to aggregate the polymer fibers (Fig. 1c). This process enables the fabrication of the desired number of polymer wires at the desired location. Subsequently, it was possible to change the wires of the other materials through metal deposition and polymer removal (Fig. 1d). These polymer wires were used as templates to replace them with wires of the desired material by depositing the desired material on the polymer wires and removing it.

### Thermal treatment

**Fig. 2:**
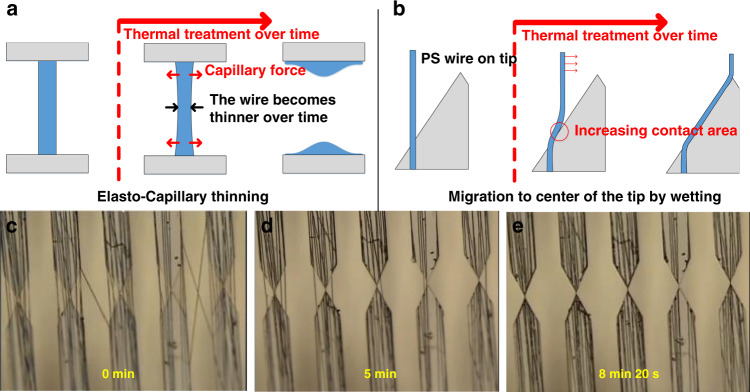
Polymer fiber aggregation mechanism during thermal treatment. **a** wetting mechanism of polymer melts (polymer fibers migrate to the center of the tip during wetting), **b** Elasto-capillary thinning of molten polymer fibers. Microscopic image of the chip with polymer fibers (**c**) before thermal treatment, **d** during thermal treatment, and **e** after thermal treatment

The migration of the polymer fiber to a specific location occurred when the thermoplastic polymer melted during the thermal treatment process (Fig. 2). The polystyrene (PS) and polyethylene oxide (PEO) we used are viscoelastic fluids that have both elastic and viscous properties when they melt. Therefore, when the suspended polymer fiber melts and becomes a liquid bridge, two mechanisms cause the migration and thinning of the polymer fiber. The first mechanism is elasto-capillary thinning^37–39^, in which the liquid bridge with elastic properties becomes thinner over time (Fig. 2a). Subsequently, the polymer fiber migrated to the center by wetting (Fig. 2b)^40–43^. Molecular kinetic theory (MKT), which may explain the migration of the fibers, was proposed by Blake and Haynes to describe the dynamics of wetting^40,41^. Here, the relationship between the dynamic contact angle *θ*_*d*_ and the contact line velocity *V* is given by

$$V = 2\kappa ^0\lambda {\mathrm{sinh}}\left( {\frac{{\gamma \left( {{\mathrm{cos}}\theta ^0 - {\mathrm{cos}}\theta _d} \right)}}{{2nk_BT}}} \right)$$

where *κ*^0^, *λ*, *n*, *k*_*B*_, *T*, *γ*, and *θ*^0^ are the jump frequency, jump length, number of adsorption sites per unit area of the substrate, Boltzmann constant, absolute temperature, liquid/vapor interfacial tension, and static contact angle, respectively. Since *γ*(cos*θ*^0^*−*cos*θ*_*d*_*)* is much larger than 2*nk*_*B*_*T*, sinh(χ) ≈ χ by linear approximation. Thus, *V* is proportional to (cosθ^0^*−*cos*θ*_*d*_), which indicates that rapid migration can be induced by placing the polymer melts to have a high *θ*_*d*_. To form a high *θ*_*d*_, a proper microstructure design was required to guide the direction of the migration of the polymer fibers to the desired location. We used simple triangular tips such that the distance between the two facing microstructures gradually decreased to the tip-to-tip distance. The dynamic contact angle of the molten polymer on the tip varies depending on the direction and is maximum in the direction to the tip end. Since the angle is extremely high (at least 150° on our designed tip), migration to the corresponding direction occurs rapidly. In general, as wetting progresses, the contact angle decreases, and the wetting speed decreases. However, in the case that the liquid bridge polymer melts on our designed tips, the same wetting occurs on the opposite side, the wire portion migrates, and the dynamic contact angle is maintained. Therefore, the migration continues until the wire reaches the tip end. The microstructure used in this study is the simplest design that can use the proposed mechanism. The gap between the tips was 5 μm, and the width of the tips was 50 μm.

For the polymer fibers to be aggregated into a single polymer wire, the migration of the fibers at desired locations and breakage of other fibers at undesired sites must occur simultaneously. Since the section in which the gap distance decreases exists only between the facing tips, migration by wetting does not occur on polymer fibers at undesired locations, and elasto-capillary thinning causes them to break. In the case of polymer fibers at the desired location, migration occurs by wetting. In addition to elasto-capillary thinning, the migration also has the effect of thinning the suspended portions of the fibers because the molten polymer remains on the migration path. Therefore, the single polymer fiber at the desired location can also be broken during thermal treatment. However, complete breakage of the polymer fibers does not occur because there are several polymer fibers at the desired location. Multiple polymer fibers are aggregated during migration to increase the thickness of the suspended portion of the wire before the individual polymer fibers become thinner and break. Eventually, the thickness of the aggregated polymer wire becomes thicker than that of individual polymer fibers before thermal treatment. If thermal treatment continued after the migration was complete, the wire became thinner and finally broke. However, the polymer wire takes more than 5 min to break; thus, it was not difficult to control the thermal treatment time to avoid breaking the wire.

In addition to the microstructure design, polymers formed by this mechanism are affected by electrospinning conditions. A characteristic of electrospinning is that the location of the electrospun fibers is random. Because of this randomness, polymer fibers may not be formed on the tip in short-term electrospinning cases. This feature can cause problems with uniformity when fabricating multiple chips simultaneously. As a result, at least 45 s of electrospinning was required to reliably fabricate polymer fibers on the tip. More polymer fibers aggregate during the thermal treatment process when more polymer fibers are generated on the tip during long-term electrospinning, resulting in thick polymer wire formations. Then, a wet process for reducing the thickness of the polymer wire was conducted. We fabricated PS wires using this mechanism. The thermal treatment was performed in an oven at 170 °C for 3 min. When the temperature was lower, the polymer fibers aggregated very slowly or failed to aggregate. The fibers aggregated and broke quickly at higher temperatures, making it difficult to stop the heating in a timely manner. In addition, we fabricated a PEO wire for microscopic observations. Because the melting point of PEO is lower than that of PS, microscopic imaging was possible when thermal treatment at a 70 °C on a hot plate was applied. Aligned PEO fibers were prepared by electrospinning two grounded electrodes (Fig. 2c). Even if the polymer fibers were not aligned, a single polymer wire remained well after thermal treatment; however, the aligned polymer fibers were prepared to identify the mechanism. Afterward, we observed the migration of the polymer fibers after placing the chip on the hot plate (Video S1). After a few minutes, the polymer fibers aggregated into a single polymer wire (Fig. 2c–e). After a longer period, the wire became thinner and finally broke. This experiment was used to confirm the mechanism and feasibility of the fabrication of other polymer materials.

### Nanowire formation

**Fig. 3:**
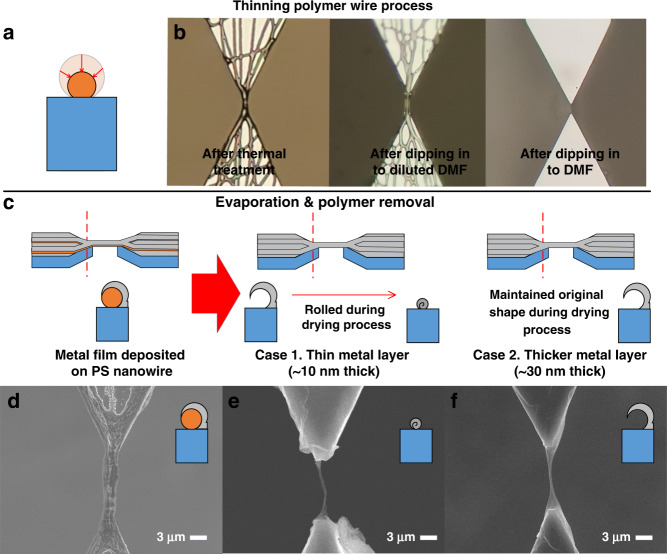
Thinning polymer wire process, evaporation, and polymer removal. **a** schematic of the process of dissolving the polymer wire for size reducing, **b** microscopic photographs during the polymer thinning process, and **c** schematic diagram of the evaporation and polymer removal process. The shell-shaped nanowire formed on a polymer wire template by evaporation. The PS wire was removed by immersing the chip in DMF. After the chip was removed, the shell-shaped 10-nm-thick nanowire was rolled during the drying process. In the case of the 30-nm-thick shell-shaped nanowire that maintained its shape of the **d** SEM image of the shell-shaped Cu nanowire on the polymer wire, **e** the SEM image of the shell-shaped 10-nm-thick nanowire was rolled, and **f** the SEM image of the 30-nm-thick shell-shaped nanowire

Using the previously described mechanism, the polymer wire was fabricated at the desired location. As mentioned above, the fabricated polymer wires were thick, ranging from 1 to 3 μm, owing to the long period of electrospinning. Metal or metal oxide nanowires that are finally formed after the material exchange process become thinner. However, to obtain thinner nanowires, we adjusted the thickness of the polymer wire, which served as a template. The polymer wire was dissolved in a suitable solvent to control the thickness (Fig. 3a). Polymer wires of 3 μm thickness were dipped into dimethylformamide (DMF) (Fig. 3b). After the DMF was completely dry, SEM measurements, which were ~500 nm thick, were taken. In this process, the thickness of the wire after drying can be controlled by adjusting certain conditions, such as the type of solvent and the time of dissolution. After controlling the thickness of the polymer wire to act as a template, the material exchange process was performed in two steps: slanted evaporation and a subsequent wet process. When the desired material is deposited through slanted evaporation, a shell-shaped wire is formed on the polymer wires (Fig. 3c). Tilting the chips during the deposition process has two advantages. The anchor between the nanowire and the substrate is formed by slanted evaporation. The area where the polymer is exposed to the solvent during the polymer removal process is large, allowing the polymer to be easily removed. The chip was immersed in DMF after deposition to remove the polymer. Because the polymer is only a few micrometers thick, it was removed quickly. Afterward, the chip was removed and dried in open air. As the solvent dries, the size of the solvent drop shrinks, exposing shell-shaped structures to the air, causing the surface tension of the solution to act on the shell-shaped nanowire. Because the surface tension is in the inward direction of the solution, it rolls the shell-shaped wires.

Shell-shaped wires are rolled by the surface tension forces of the solvents. When the surface tension force is insufficient, shell-type wires remain in shape without being rolled. By adjusting the thickness of the evaporation material, two types of wires can be fabricated: one maintained shell form and one rolled form. When using DMF as the solvent, it was confirmed that the evaporation thickness of 30 nm was maintained in the shell form and that the evaporation thickness of the 10 nm deformed to the rolled form (Fig. 3d–f). The rolled form is a nanowire shape, and the shell form is shaped like a bent nanoribbon that is 30 nm thick and several hundreds of nanometers wide. Between these two shapes, we focused on the nanowire to further demonstrate the fabrication limit of the smallest thicknesses of the nanowires.

### Characteristics and applications of the nanowires

**Fig. 4:**
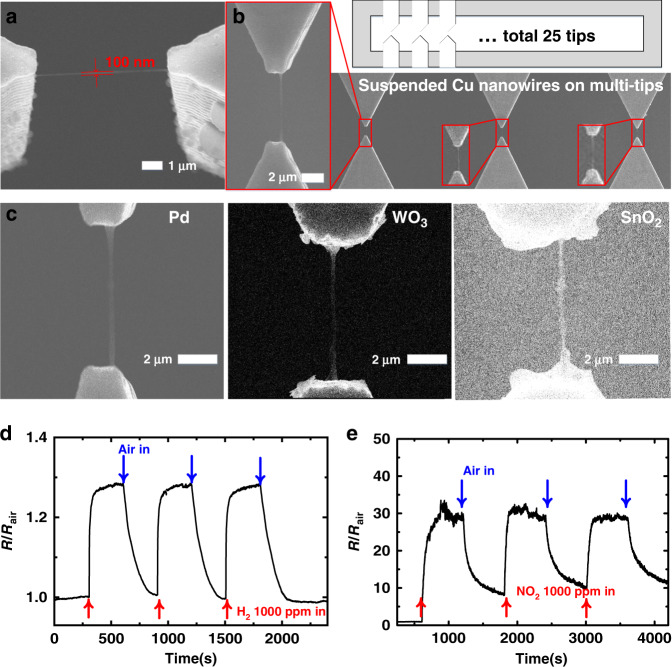
Characteristics of the fabricated nanowires. **a** SEM image of sub-100-nm Cu nanowire. **b** SEM images of Cu nanowires formed on multi-tips. The microstructure was designed to have 25 tips. **c** SEM images of Pd, WO_3_, and SnO_2_ nanowires. **d** Resistance response of the Pd nanowire gas sensor for 1000 ppm air-diluted H_2_ gas. **e** Resistance response of the WO_3_ nanowire gas sensor for 1000 ppm air-diluted NO_2_ gas

To confirm the characteristics of the developed method, we conducted several additional experiments. A nanowire with a length of 20 μm was fabricated by changing the design of the microstructure (Fig. S1a). For different gap distances, the detailed experimental variables, such as the electrospinning time, thermal treatment time and thickness of the evaporated material, should be adjusted differently. The minimum thicknesses of the nanowires that can be fabricated was measured to be sub-100 nm (Fig. S1b). The thickness of the nanowires can be controlled by dissolving the aggregated polymer wire and the time of electrospinning (Fig. 4a). The sub-100-nm nanowires were fabricated under the condition that electrospinning was performed for 180 s, and the aggregated polymer wire was dipped in undiluted DMF for thickness control. A shorter electrospinning time seems to fabricate thinner nanowires; however, the exact thickness of the nanowire could not be measured by SEM. This is because the nanowires were broken when observed with SEM electron beams (Fig. S2).

In addition, to demonstrate that batch fabrication is possible, a platform with 25 multiple tips was designed. The same fabrication method was conducted on the platform, and nanowires were formed on 22 of the 25 tips (Fig. 4b). Nanowire fabrication of various materials was also conducted. Pd and Cu nanowires were fabricated from metal nanowires. In addition, WO_3_ and SnO_2_ nanowires, which are metal oxides with low ductility compared to metals, were successfully fabricated (Fig. 4c). We believe that other metal and metal oxide nanowires can be fabricated. Among the many materials that can be deposited by evaporation, palladium, tungsten oxide, and tin oxide were chosen as nanowire materials because they are gas-sensing materials. The suspended nanowires of gas-sensing materials can be used directly as gas sensors by adding simple electrical connections.

A gas-sensing experiment was conducted to determine the electrical characteristics of the nanowires. Simple wire bonding was performed on the fabricated nanowires for use as a gas sensor. The palladium nanowire gas sensor was exposed to 1000 ppm H_2_ gas for 5 min, and the resistance of the nanowire reached 1.28 times its initial resistance. The sensor was then exposed to airflow for 5 min (Fig. 4d). The tungsten oxide nanowire was exposed to 1000 ppm NO_2_. This experiment was repeated for three cycles with 15 min of gas exposure and 15 min of recovery time (Fig. 4e). The tungsten oxide nanowires worked well as gas sensors, with a response of over 30. In addition, this nanowire gas sensor detected 20 ppm NO_2_ (Fig. S3). Originally, tungsten oxide nanowires failed to react with NO_2_ gas at room temperature^8,44^; however, it was confirmed that the self-heating effect occurred at a low voltage of 1 V to react with the gas. Because it is a suspended nanowire, the heat transfer to the substrate occurs only at both anchored ends, resulting in self-heating at a low voltage of 1 V. The response values for these two gas sensors are similar to those of suspended nanowires with the same material^31,44^. These gas-sensing experiments confirmed that the nanowires had no problems with their electrical characteristics and were available as application devices.

## Discussion

A novel method for the fabrication of suspended nanowires has been developed. The realignment of polymers during thermal treatment is the key mechanism of this method, and the shape of the nanowires can be controlled by adjusting the electrospinning conditions and designing the microstructure. The fabricated nanowires are completely controlled in terms of the material, number, and location. Regarding thickness, it was possible to fabricate thin nanowires of <100 nm. By this method, nanowires can be easily fabricated on the microstructure. Therefore, it is a convenient method for fabricating nanowire-integrated MEMS devices. As an example, a simple-structured gas sensor was fabricated, and a performance experiment was conducted. We believe that this method can be used to fabricate various nanowire-integrated MEMS devices, including gas sensors.

## Materials and methods

### Electrospinning

A solution of 20 wt% PS in DMF was prepared. The prepared solutions were extracted using a syringe pump. A voltage of 9.4 kV was applied to the needle and two ground electrodes using a high-voltage generator. A 34-gauge needle was used, and the distance between the needle tip and the sample was maintained at 10 cm.

### Thermal treatment

Oven and hot plates were used as devices to apply heat. A hot plate was used to confirm the migration of the polymer fibers directly using a microscope. The chip with PEO fibers was placed on a hot plate that was preheated to 80 °C. After confirming that the polymer fibers aggregated to a single wire through a microscope, the chip was removed. The oven was mainly used, except during the experiments, to investigate the mechanism. The chips with PEO and PS fibers were placed in a preheated oven at 80 °C and 170 °C, respectively, for 3 min.

### Gas sensing

The sensor was tested in a chamber by exposing it to air-diluted H_2_ or NO_2_ gas at room temperature. Air-diluted H_2_ or NO_2_ gas flowed into the chamber, and the resistance change was monitored using a source meter (2400 SourceMeter, Keithley). The gas flow rate and flow time were controlled using a mass flow controller connected to a laptop.

## Supplementary information

Supplementary Information

supplementary video Oh

## References

[1] Brabazon, D. et al. *Commercialization of Nanotechnologies–A Case Study Approach* (Springer, 2017).

[2] Kong W (2019). Path towards graphene commercialization from lab to market. Nat. Nanotechnol..

[3] Brenner, A., Bornschlegel, B. & Finger, J. *Nanoengineering: Fabrication, Properties, Optics, and Devices XV. 107300H* (International Society for Optics and Photonics). (2018).

[4] Haroun A (2021). Progress in micro/nano sensors and nanoenergy for future AIoT-based smart home applications. Nano Express.

[5] Kalsoom T, Ramzan N, Ahmed S, Ur-Rehman M (2020). Advances in sensor technologies in the era of smart factory and industry 4.0. Sensors.

[6] Zhu J (2020). Development trends and perspectives of future sensors and MEMS/NEMS. Micromachines.

[7] Mishra, M. K., Dubey, V., Mishra, P. & Khan, I. MEMS technology: a review. *J. Eng. Res. Rep.* **4**, 1–24 (2019).

[8] Yuan K (2020). Fabrication of a micro-electromechanical system-based acetone gas sensor using CeO2 Nanodot-Decorated WO3 nanowires. ACS Appl. Mater. interfaces.

[9] Nag A, Mitra A, Mukhopadhyay SC (2018). Graphene and its sensor-based applications: a review. Sens. Actuat. A: Phys..

[10] Ngoc TM (2019). Effective design and fabrication of low-power-consumption self-heated SnO2 nanowire sensors for reducing gases. Sens. Actuat. B: Chem..

[11] Zang X, Zhou Q, Chang J, Liu Y, Lin L (2015). Graphene and carbon nanotube (CNT) in MEMS/NEMS applications. Microelectron. Eng..

[12] Yang D (2019). Gas sensor by direct growth and functionalization of metal oxide/metal sulfide core–shell nanowires on flexible substrates. ACS Appl. Mater. interfaces.

[13] Hsueh T-J, Peng C-H, Chen W-S (2020). A transparent ZnO nanowire MEMS gas sensor prepared by an ITO micro-heater. Sens. Actuat. B: Chem..

[14] Wang Y (2022). A high-performance ethanol gas sensor based on Ce-doped SnO2 nanomaterials prepared by the Pechini method. Mater. Sci. Semicond. Process..

[15] Meng G (2016). Nanoscale thermal management of single SnO2 nanowire: pico-joule energy consumed molecule sensor. ACS Sens..

[16] Park M-S, Kang Y-M, Dou S-X, Liu H-K (2008). Reduction-free synthesis of carbon-encapsulated SnO 2 nanowires and their superiority in electrochemical performance. J. Phys. Chem. C.

[17] Zhou C, Zheng K, Chen P-P, Lu W, Zou J (2017). Unexpected formation of a hierarchical structure in ternary InGaAs nanowires via “one-pot” growth. Nanoscale.

[18] Baek D-H, Choi J, Kim J (2019). Fabrication of suspended nanowires for highly sensitive gas sensing. Sens. Actuat. B: Chem..

[19] Lee K, Baek D-H, Choi J, Kim J (2018). Suspended CoPP-ZnO nanorods integrated with micro-heaters for highly sensitive VOC detection. Sens. Actuat. B: Chem..

[20] Choi J, Kim J (2009). Highly sensitive hydrogen sensor based on suspended, functionalized single tungsten nanowire bridge. Sens. Actuat. B: Chem..

[21] Lim Y, Lee Y, Heo J-I, Shin H (2015). Highly sensitive hydrogen gas sensor based on a suspended palladium/carbon nanowire fabricated via batch microfabrication processes. Sens. Actuat. B: Chem..

[22] Huh J, Park J, Kim GT, Park JY (2011). Highly sensitive hydrogen detection of catalyst-free ZnO nanorod networks suspended by lithography-assisted growth. Nanotechnology.

[23] Jian S (2022). Enhanced visible light photocatalytic efficiency of La-doped ZnO nanofibers via electrospinning-calcination technology. Adv. Powder Mater..

[24] Shui J, Li JC (2009). Platinum nanowires produced by electrospinning. Nano Lett..

[25] George D (2020). Fabrication of patterned graphitized carbon wires using low voltage near-field electrospinning, pyrolysis, electrodeposition, and chemical vapor deposition. Microsyst. Nanoeng..

[26] Lei T (2018). New insight into gap electrospinning: toward meter-long aligned nanofibers. Langmuir.

[27] Cai X (2017). Electrospinning of very long and highly aligned fibers. J. Mater. Sci..

[28] Chang G (2018). Functional carbon nanofibers with semi‐embedded titanium oxide particles via electrospinning. Macromol. Rapid Commun..

[29] Lee WS, Park Y-S, Cho Y-K (2014). Hierarchically structured suspended TiO2 nanofibers for use in UV and pH sensor devices. ACS Appl. Mater. Interfaces.

[30] Huang S, Liu Y, Guo CF, Ren Z (2017). A highly stretchable and fatigue‐free transparent electrode based on an in‐plane buckled au nanotrough network. Adv. Electron. Mater..

[31] Liang J (2022). Constructing a high-density thermally conductive network through electrospinning–hot-pressing of BN@ PDA/GO/PVDF composites. ACS Appl. Polym. Mater..

[32] Thiha A (2018). All-carbon suspended nanowire sensors as a rapid highly-sensitive label-free chemiresistive biosensing platform. Biosens. Bioelectron..

[33] Salazar A, Cardenas-Benitez B, Pramanick B, Madou MJ, Martinez-Chapa SO (2017). Nanogap fabrication by Joule heating of electromechanically spun suspended carbon nanofibers. Carbon.

[34] Coşkun M, Ombaba MM, Dumludağ F, Altındal A, Islam MS (2018). Bridged oxide nanowire device fabrication using single step metal catalyst free thermal evaporation. RSC Adv..

[35] Li Y (2022). Catalyst electrodes with PtCu nanowire arrays in situ grown on gas diffusion layers for direct formic acid fuel cells. ACS Appl. Mater. interfaces.

[36] Lee JS, Choi KW, Yoo JY, Jo MS, Yoon JB (2020). Realization of nanolene: a planar array of perfectly aligned, air‐suspended nanowires. Small.

[37] Anna SL, McKinley GH (2001). Elasto-capillary thinning and breakup of model elastic liquids. J. Rheol..

[38] Kolte MI, Szabo P (1999). Capillary thinning of polymeric filaments. J. Rheol..

[39] Neelakantan R (2020). The effect of end-plate wetting and unpinned contact lines on the filament thinning of strain hardening fluids. Phys. Fluids.

[40] Blake TD, Haynes J (1969). Kinetics of liquidliquid displacement. J. Colloid Interface Sci..

[41] Pucci MF (2020). Dynamic wetting of molten polymers on cellulosic substrates: model prediction for total and partial wetting. Front. Mater..

[42] Vera J (2017). Wetting of polymer melts on coated and uncoated steel surfaces. Appl. Surf. Sci..

[43] Bertola V (2013). Dynamic wetting of dilute polymer solutions: the case of impacting droplets. Adv. Colloid Interface Sci..

[44] Hoa ND, El-Safty SA (2011). Gas nanosensor design packages based on tungsten oxide: mesocages, hollow spheres, and nanowires. Nanotechnology.

