# Supplementary material for: Formation of sub-100-nm suspended nanowires with various materials using thermally adjusted electrospun nanofibers as templates
Source: Microsyst Nanoeng. 2023 Feb 17;9:15. doi: 10.1038/s41378-022-00459-y (PMC9935917; doi:10.1038/s41378-022-00459-y)
Supplement: Supplementary file 1 — Supplementary Information [file 41378_2022_459_MOESM1_ESM.docx]

**Formation of Sub-100 nm Suspended Nanowires with Various Materials Using Thermally Adjusted Electrospun Nanofibers as Templates**

Yongkeun Oh^†‡^, Dae-Sung Kwon^†‡^, Eunhwan Jo^†^, Yunsung Kang ^†^, Sangjun Sim ^†^, and Jongbaeg Kim^†*^

^†^School of Mechanical Engineering, Yonsei University, 50 Yonsei-ro, Seodaemun-gu, Seoul 03722, Republic of Korea

‡These authors contributed equally.

*E-mail: kimjb@yonsei.ac.kr (J.K.).

**Supplementary Information.**

Figure S1. SEM image of a (a) 20-μm-long Pd nanowire and a (b) sub-100-nm-thick Pd nanowire (docx)

Figure S2. SEM images of Pd nanowires broken by the e-beam during SEM (docx)

Figure S3. Resistance change in the WO_3_ nanowire gas sensor when exposed to 20 ppm NO_2_ gas (docx)

Supplementary Video 1. Aggregation of polymer fibers during the thermal process observed under a microscope camera (MP4)


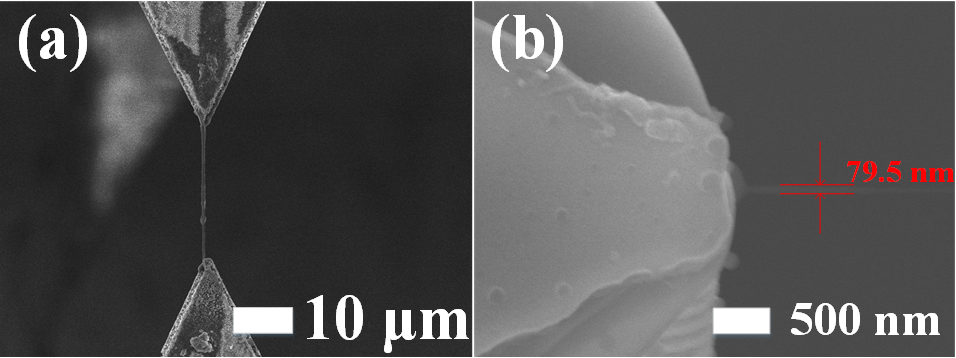


**Figure S1.** SEM image of (a) 20-μm-long Pd nanowire (b) sub-100-nm-thick Pd nanowire The smallest thickness of the nanowires that can be measured by SEM was approximately 80 nm.

**Figure S2.** (a) SEM image of a 50 nm thick Pd nanowire broken by e beam during SEM. Although nanowires that are thinner than 100 nm can be fabricated, they are difficult to measure with SEM. (b) Fabricated Pd nanowire was observed by SEM at 5000 magnification; (c) SEM image of the broken Pd nanowire. The nanowire observed in (b) was isolated while changing the magnification of the SEM from 5000 to 8500.


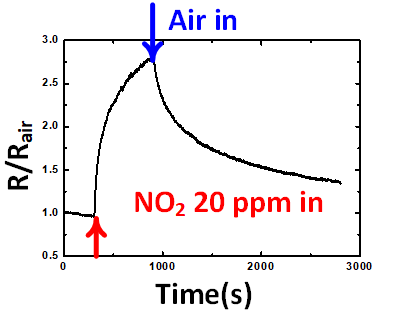


**Figure S3.** Resistance change in the WO_3_ nanowire gas sensor when exposed to 20 ppm NO_2_ gas for 10 min
